# Perceived ageism and suicidal ideation among Chinese older adults: the mediating role of rumination and self-perceived aging

**DOI:** 10.3389/fpsyt.2026.1747979

**Published:** 2026-04-10

**Authors:** Liting Fu, Yadong Zhou, Yumo Zhang

**Affiliations:** 1 Anhui University of Chinese Medicine, Hefei, China; 2 Kaifeng Traditional Chinese Medicine Hospital, Kaifeng, China

**Keywords:** older adults, perceived ageism, rumination, self-perceived aging, suicidal ideation

## Abstract

**Objective:**

To examine the impact of perceived ageism on suicidal ideation among Chinese older adults, and the chained mediating role of rumination and self-perceived aging therein.

**Methods:**

A questionnaire survey was conducted among 326 older adults using the Perceived Ageism Scale, Ruminative Responses Scale, Brief Ageing Perceptions Questionnaire and Self-rating Idea of Suicide Scale.

**Results:**

(1) Correlation analysis revealed significant positive correlations among perceived ageism, rumination, self-perceived aging, and suicidal ideation; (2) Rumination and self-perceived aging partially mediated and chain-mediated the relationship between perceived ageism and suicidal ideation among older adults.

**Conclusion:**

Perceived ageism not only directly increases the risk of suicidal ideation among older adults but also indirectly contributes to it by triggering rumination and reinforcing negative self-perceptions of aging. More importantly, rumination may lead individuals to internalize external ageist experiences, thereby fostering a more negative view of aging; together, these factors synergistically amplify the risk of suicidal ideation. These findings highlight the need for early psychological intervention to mitigate the impact of perceived ageism on suicidal ideation through the pathways of rumination and self-perceived aging.

## 1 Introduction

Population aging has become a major challenge in global public health. As the country with the world’s largest elderly population, China faces particularly significant impacts. According to official data from the National Bureau of Statistics of China, by the end of 2020, there were 264 million people aged 60 and above in China, with the elderly population projected to reach 350 million by 2030 (1). Elderly individuals constitute a high-risk group for suicide (2), and against the backdrop of an aging society, elderly suicide deaths may rise accordingly (3). Suicidal ideation primarily refers to thoughts, attempts, and plans preceding suicide attempts, serving as a significant predictor of suicidal behavior (4). A meta-analysis examining the epidemiological characteristics of suicidal ideation among 79,861 Chinese older adults revealed a prevalence rate of 15.59% between 2011 and 2020—double the 7.85% rate observed from 2001 to 2010 (5). Furthermore, the lifetime prevalence of suicidal ideation reached 9.2% (6), posing a severe threat to the health and well-being of older adults. In summary, examining the influencing factors and mechanisms of suicidal ideation among older adults holds significant social and practical importance for preventing and intervening in suicidal ideation and suicide among this population.

Ageism, a common source of psychosocial stress, shares similar projection mechanisms with racial and gender discrimination (7), It is frequently used to denote discrimination against older individuals (8). Perceived ageism, a manifestation of age discrimination, refers to older adults’ subjective experience of negative treatment and disadvantageous distinctions based on ages (9). Within social structures, older adults are typically defined by society, others, and themselves as a vulnerable group, believed to be more susceptible to age discrimination (10). Moreover, perceived ageism increases with advancing age (11). As a common stressor triggering adaptive mechanisms in older adults, perceived ageism may undermine psychosocial resources such as interpersonal support and sense of control, thereby weakening their capacity to cope with stress. This leads to lower levels of well-being and higher levels of psychological distress (12). Furthermore, research confirms that older adults who perceive ageism exhibit stronger suicidal ideation and are more likely to progress from suicidal thoughts to suicide attempts (13, 14).

Rumination is a maladaptive cognitive pattern where individuals repeatedly focus on the event itself, its causes, and consequences when facing stressful situations, without taking action to alter their current predicament (15). Perceived discrimination can trigger rumination (16). For instance, Wang and Borders (17) found that among sexual minority groups, rumination levels increased alongside heightened perceived discrimination. Therefore, perceived ageism, as a form of prejudice, may be positively correlated with rumination. Furthermore, the stress-sensitization model of suicide posits that perceived ageism, as a stressor, may interact with individual susceptibility factors like rumination to induce suicidal behavior (18). Le et al. (19) research also confirmed a significant positive correlation between rumination and suicidal ideation, with rumination further predicting lifetime history of suicide attempts. Previous studies have similarly demonstrated that rumination mediates the relationship between perceived discrimination (e.g., perceived racial discrimination) and negative mental health outcomes (e.g., depression) (20).

Self-Perceived Aging (SPA) is defined as older adults’ beliefs or evaluations regarding their own aging (21). The theory of stereotype embodiment suggests that individuals’ aging self-perceptions, constructed through the internalization of societal age stereotypes throughout life, influence their functioning and health (22). Numerous studies have validated this theory. For instance, Coudin and Alexopoulos (23) and Marquet et al. (24) found that heightened levels of perceived negative age stereotypes and age discrimination predict greater susceptibility to developing negative self-perceptions of aging. This, in turn, undermines subjective health and self-esteem, elevates negative emotions such as loneliness, anxiety, and depression (25) and may even trigger death anxiety and suicidal ideation (26). Kim et al. (8) further confirmed that self-perceived aging mediates the relationship between perceived ageism and negative psychological outcomes. The Perseverative Cognition Hypothesis suggests that rumination may prolong and amplify the negative consequences of perceived age discrimination, such as negative self-perceptions of aging (26). Furthermore, Dutt et al. (27) confirmed in a 4.5-year longitudinal study that rumination positively predicts negative aging cognitions among older adults. Specifically, older adults exhibiting high levels of rumination selectively attend to and dwell on issues related to exclusion and unfair treatment stemming from discrimination, potentially exacerbating negative self-aging beliefs arising from age discrimination.

**Figure 1:**
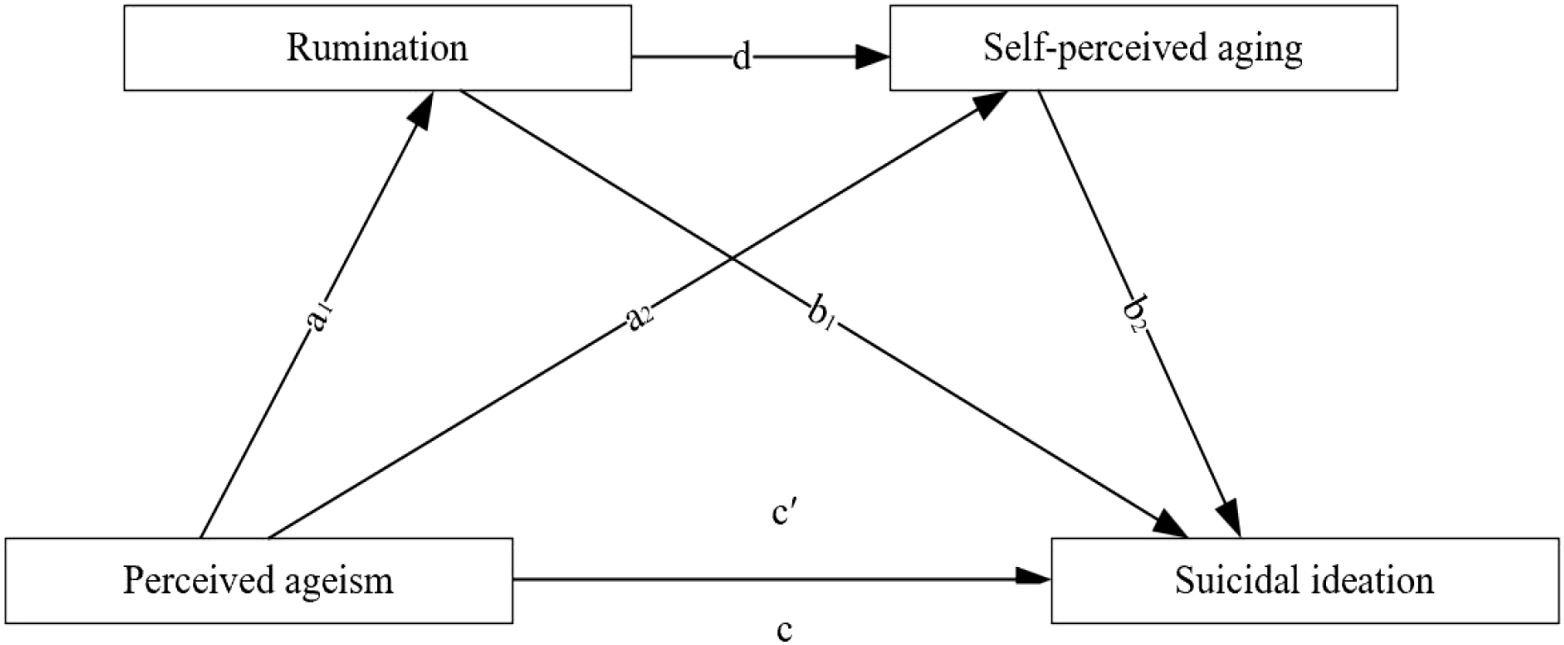
A chained mediation model illustrating how perceived ageism influences suicidal ideation through rumination and perceived self-aging.

In summary, this study integrates the Stereotype Manifestation Theory, the Stress-Vulnerability Model of Suicide, and the Persistent Cognitive Hypothesis to construct a chained mediation model (see **Figure 1**). This model aims to examine the combined effects of perceived ageism, rumination, and self-perceived aging on suicidal ideation among Chinese older adults. It further seeks to reveal the mechanisms through which perceived ageism influences suicidal ideation, as well as the threatening roles of rumination and self-perceived aging in the formation of suicidal ideation. Specific hypotheses are as follows:

Research Hypothesis H1: Perceived ageism positively predicts suicidal ideation among older adults.
Research Hypothesis H2: Rumination may mediate the relationship between perceived ageism and suicidal ideation among older adults.
Research Hypothesis H3: Self-perceived aging may mediate the relationship between perceived ageism and suicidal ideation among older adults.
Research Hypothesis H4: Rumination and self-perceived aging may exert a chain mediation effect between perceived ageism and suicidal ideation among older adults.

## 2 Materials and methods

### 2.1 Study design and sample

This study was conducted in 2020, surveying 400 individuals aged 60 and above across six elderly care institutions in a city in northeastern China. Prior to the survey, trained psychology graduate students informed all participants about the study’s purpose, use, anonymity, and right to withdraw at any time, and obtained signed informed consent forms. For participants with lower educational attainment or reading difficulties, the principal investigator administered the questionnaire via one-on-one oral interviews. After excluding invalid questionnaires, 326 valid responses were retained. The sample comprised 168 males and 158 females, with a mean age of 67.83 ± 5.54 years.

### 2.2 Measures

#### 2.2.1 Perceived ageism scale

This study adopted the assessment method from the European Survey on Ageism Prevalence (26). The scale consists of three items rated on a 5-point Likert scale (0=never to 4=always), yielding a total score ranging from 0 to 12. A score of ≥1 indicates the presence of ageism, with higher scores reflecting greater perceived ageism. The internal consistency (Cronbach’s α) of the scale in this study was 0.875.

#### 2.2.2 Ruminative responses scale

This scale was introduced into China by Han and Yang (28). It consists of 22 items covering three dimensions—symptomatic rumination, compulsive thinking, and reflective rumination—and is rated on a 4-point Likert scale (1=occasionally to 4=always). Total scores range from 22 to 88. A score ≤35 indicates low-level rumination; a score between 36 and 40 indicates moderate rumination; and a score ≥41 indicates high-level rumination (29). The internal consistency (Cronbach’s α) of the scale in this study was 0.952.

#### 2.2.3 Brief ageing perceptions questionnaire

This scale was revised by Hu et al. (30) and consists of 17 items covering five dimensions: chronic timeline, positive consequences, positive control, negative consequences and control, and emotional representation. It uses a 5-point Likert scale (1=strongly disagree to 5=strongly agree). Total scores range from 17 to 85, with higher scores indicating more negative self-perceptions of aging. The internal consistency (Cronbach’s α) of the scale in this study was 0.802.

#### 2.2.4 Self-rating idea of suicide scale

This scale was developed by Zhang et al. (31) and consists of 26 items across four dimensions: Hopelessness (12 items), Optimism (5 items), Sleep (4 items), and Concealment (5 items). It uses a dichotomous scoring system (Yes=1 point, No=0 points). The total suicidal ideation score is calculated by summing the scores from the Hopelessness, Optimism, and Sleep dimensions, yielding a total score ranging from 0 to 21. Higher total scores indicate stronger suicidal ideation. The internal consistency (Cronbach’s α) of the scale in this study was 0.856.

### 2.3 Statistical analysis

This study used SPSS 24.0 software for normality testing, descriptive statistics, and correlation analyses. Specifically: First, the Shapiro–Wilk test was employed to assess the normality of all included variables. Results showed that scores for Perceived Ageism (W = 0.541, *p* < 0 0.001), Rumination (W = 0.888, *p* < 0.001), and Suicidal Ideation (W = 0.900, *p* < 00.001) significantly deviated from normality, whereas Self-Perceived Aging met the assumption of normality (W = 0.992, *p* = 0.078). Second, non-normally distributed continuous variables were described using the median (minimum, maximum), denoted as M (min, max), while normally distributed continuous variables were reported as mean ± standard deviation (*M* ± *SD*). Third, given the presence of non-normal variables, Spearman’s rank-order correlation analysis was used to examine bivariate associations among all study variables. Finally, due to the non-normality of key variables, a bias-corrected bootstrap procedure with 5,000 resamples was implemented via Hayes’ PROCESS Model 6 to estimate direct and indirect effects. The bootstrap method does not assume normality of the sampling distribution and is widely recommended for mediation analyses involving skewed or zero-inflated data. A 95% confidence interval (CI) was computed for each effect; paths whose CIs did not include zero were considered statistically significant at the *p* < 0.05 level.

## 3 Results

### 3.1 Characteristics of participants

Descriptive statistics and Spearman rank correlation analysis revealed (see **Table 1**) that perceived ageism, rumination, self-perceived aging, and suicidal ideation were significantly and positively correlated with each other. This pattern meets the statistical requirements for further examining chain mediation between perceived ageism and suicidal ideation.

**Table 1:** Descriptive statistics and correlation analysis results for each variable.

Variant	Description	1	2	3	4
	*M (min, max)*				
1 PA	0(0,9)	1			
2 R	31(22,83)	0.421***	1		
3 SI	4(0,19)	0.316***	0.553***	1	1
	*M* ± SD				
4 SPA	48 ± 10.20	0.337***	0.526***	0.439***	

PA, Perceived ageism; R, Rumination; SPA, Self-perceived aging; SI, Suicidal ideation.

***indicates *P* < 0.001, **indicates *P* < 0.01, and *indicates *P* < 0.05.

### 3.2 The chain mediating model analysis

Standardized the variables in the study. With perceived ageism as the independent variable, suicidal ideation as the dependent variable, and rumination and self-perceived aging as mediating variables, the results of the mediating effect test are shown in **Table 2**. The total effect of perceived ageism on suicidal ideation was significant (*β* = 0.367, *P* < 0.01), and the direct effect was also significant (*β* = 0.097, *P* < 0.05). As perceived ageism increased, rumination levels also increased (*β* = 0.388, *P* < 0.01), and self-perceived aging levels also increased (*β* = 0.230, *P* < 0.01); Rumination was positively associated with both self-perceived aging (*β* = 0.391, *P* < 0.01) and suicidal ideation (*β* = 0.522, *P* < 0.01); self-perceived aging was also positively associated with suicidal ideation (*β* = 0.178, *P* < 0.01).

**Table 2:** Analysis of chained intermediary model.

Regression equation	Equation 1: SI		Equation 2: R		Equation 3: SPA		Equation 4: SI	
	*β*	*t*	*β*	*t*	*β*	*T*	*β*	*t*
PA	0.367	7.107***	0.388	7.571***	0.230	4.482***	0.097	2.108*
R					0.391	7.610***	0.522	10.772***
SPA							0.178	3.679***
*R^2^*	0.135		0.150		0.276		0.455	
*F*	50.508***		57.319***		61.459***		89.646***	

PA, Perceived ageism; R, Rumination; SPA, Self-perceived aging; SI, Suicidal ideation, ***indicates *P* < 0.001, **indicates *P* < 0.01, and *indicates *P* < 0.05.

**Figure 2:**
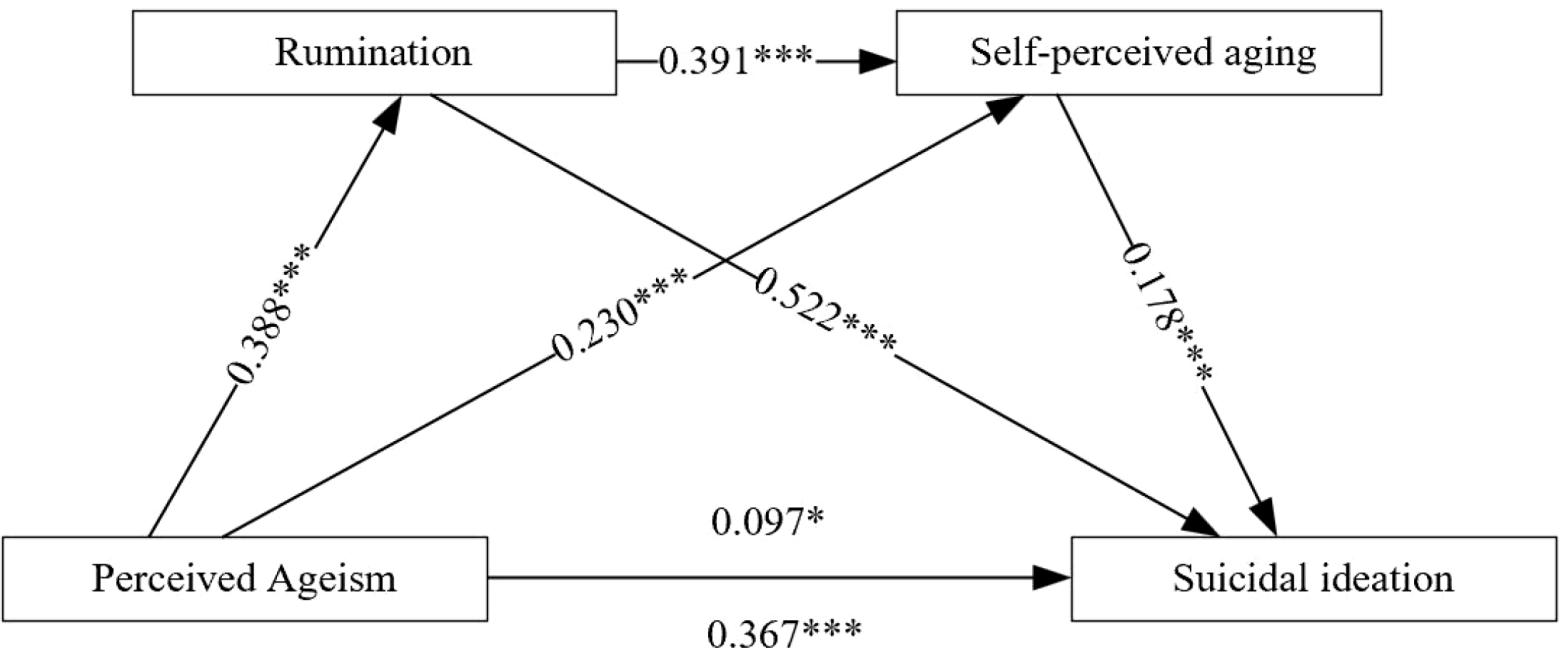
Chain mediation model of Perceived ageism and suicidal ideation through rumination and self-perceived aging. Path coefficients are standardized coefficients. ***indicates *P* < 0.001, **indicates *P* < 0.01, and *indicates *P* < 0.05.

The results of the mediation analysis are presented in **Table 3**. The findings indicate that the Bootstrap 95% confidence intervals for the indirect effects all exclude zero, confirming that rumination and self-perceived aging exhibit a statistically significant chained mediating association in the relationship between perceived ageism and suicidal ideation. The mediation effect value is 0.270, accounting for 73.57% of the total effect (0.367). This mediating effect comprises three pathways: (1) Indirect Effect 1 (0.202): Perceived ageism → Rumination → Suicidal ideation; (2) Indirect Effect 2 (0.041): Perceived ageism → Self-perceived aging → Suicidal ideation; (3) Indirect Effect 3 (0.027): Perceived ageism → Rumination → Self-perceived aging → Suicidal ideation. Indirect Effects 1, 2, and 3 accounted for 55.04%, 11.17%, and 7.36% of the total effect, respectively (see **Table 2**, **Figure 2**).

**Table 3:** Total effect, direct effect and indirect effect values.

Effect	Trails	Efficiency value	Boot SE	95%*CI*	Effect size(%)
Direct effect	PA→SI	0.097	0.046	[0.007,0.187]	26.43%
Indirect effect	PA→R→SI	0.202	0.034	[0.142,0.275]	55.04%
	PA→SPA→SI	0.041	0.015	[0.015,0.072]	11.17%
	PA→R→SPA→SI	0.027	0.010	[0.011,0.049]	7.36%
Total indirect effect		0.270	0.039	[0.199,0.355]	73.57%
Total effect		0.367	0.052	[0.266,0.469]	

PA, Perceived ageism; R, Rumination; SPA, Self-perceived aging; SI, Suicidal ideation.

***indicates *P* < 0.001, **indicates *P* < 0.01, and *indicates *P* < 0.05.

## 4 Discussion

This study deepens our understanding of the association between perceived ageism and suicidal ideation among institutionalized older adults in China by constructing a chained mediation model involving rumination and self-perceived aging. The findings confirm that perceived ageism is directly associated with suicidal ideation among Chinese older adults living in long-term care facilities. It is also indirectly associated with suicidal ideation through the independent mediating roles of rumination and self-perceived aging, respectively, as well as through their chained mediating pathway (rumination → self-perceived aging). This study is the first to integrate rumination, self-perceived aging, perceived ageism, and suicidal ideation among institutionalized older adults into a chain mediation model to elucidate how perceived ageism influences suicidal ideation in this vulnerable population. The findings provide theoretical and empirical support for policy and clinical interventions tailored to older adults in residential care settings, aimed at preventing and addressing suicidal ideation.

### 4.1 Score levels of perceived ageism, rumination, self-perceived aging, and suicidal ideation among institutionalized older adults

First, this study found that the total score for perceived ageism among institutionalized Chinese older adults was 0 (0, 9), which is relatively low compared to the scale’s midpoint of 6.0. This finding aligns with Cheng et al. (32). In China, filial piety culture is highly valued, which emphasizes societal respect, tolerance, and care for older individuals, thereby reducing older adults’ exposure to ageism—particularly among those in structured care environments where staff are trained in elder respect; Second, the total rumination score among older adults in long-term care facilities was 31 (22, 83), also lower than the scale’s midpoint of 44.0, consistent with Kothari et al. (29). This suggests that participants exhibited only mild, occasional ruminative tendencies rather than pathological rumination—possibly due to the routine structure and emotional support available in institutional settings; Third, the mean score for self-perceived aging was 48.00 ± 10.20, slightly above the scale’s midpoint of 42.5. However, this result is lower than Tan et al. (33) findings among rural older adults (54.63 ± 8.41) and consistent with Luo et al. (34). In recent years, accelerated social transformation in China has led to a growing number of left-behind older adults in rural areas. Compared with their rural counterparts, older adults residing in institutional care settings have more frequent peer interactions and broader social networks within the facility, enabling them to receive greater emotional support when facing stress or negative emotions—thereby mitigating negative perceptions of aging; Finally, the total suicidal ideation score was 4 (0, 19), notably lower than the scale’s midpoint of 10.5, consistent with Fu et al. (35)’s findings in Chinese older adults. Participants in this study were residents of long-term care facilities, where greater opportunities for social engagement and wider social circles help alleviate loneliness and other negative emotions, thus contributing to reduced suicidal ideation—a protective factor particularly relevant to institutionalized older adults.

### 4.2 Relationship between perceived ageism and suicidal ideation among institutionalized older adults

This study found a significant positive association between perceived ageism and suicidal ideation among institutionalized older adults, consistent with prior research (13, 36). Notably, the overall level of perceived ageism in this sample was low, suggesting that even rare or mild experiences of ageism may exert adverse psychological effects, particularly among vulnerable populations such as institutionalized older adults. Objectively, age-related declines in physical functioning and social roles may lead older adults to be categorized—or to perceive themselves—as members of low-status social groups, heightening their sensitivity to age-related cues. In China, although the cultural norm of filial piety continues to emphasize respect for older adults, its practical enactment has been weakened by the rise of individualistic values. This shift may render institutionalized older adults—especially those who already feel burdensome due to their dependence on care—more psychologically vulnerable to even occasional incidents of ageism (37). Consequently, the internalization of such experiences may erode their will to live and increase the risk of suicidal ideation (13, 36, 37).

### 4.3 The mediating role of rumination in the association between perceived ageism and suicidal ideation among institutionalized older adults

The findings of this study also reveal that rumination partially mediates the relationship between perceived ageism and suicidal ideation among institutionalized Chinese older adults. Specifically, perceived ageism is not only directly associated with older adults’ suicidal ideation but also indirectly linked to it through rumination. When residents of care facilities encounter negative comments or behaviors related to their age in real life (such as “being the subject of jokes about the elderly”), it prompts them to perceive themselves as being in a threatening and hostile environment. This may diminish their sense of control over their surroundings, trigger maladaptive cognitions, and lead them into a vicious cycle of passive rumination and fixation on the discrimination itself, along with the resulting negative emotions such as shame and feelings of uselessness (38–40). The rumination triggered by age discrimination further increases negative thoughts, diminishes problem-solving abilities, and heightens the risk of suicidal ideation (18).

### 4.4 The mediating role of self-perceived aging in the association between perceived ageism and suicidal ideation among institutionalized older adults

Furthermore, self-perceived aging partially mediates the relationship between perceived ageism and suicidal ideation among Chinese older adults in institutional care. In other words, perceived ageism is directly associated with suicidal ideation among older adults and is also indirectly linked to it through self-perceived aging. According to the looking-glass self, others’ evaluations and feedback play a crucial role in shaping an individual’s self-concept (41). This implies that institutionalized older adults may internalize others’ negative age-based evaluations of them—such as “older people are useless, dependent on others, and a burden on society”—leading to lower self-evaluations and negative self-perceptions of aging, such as “I am worthless” or “I am a burden to others.” As proposed by interpersonal theories of suicide, the perception that “death is more valuable than life” often stems from individuals feeling useless and believing they impose burdens or stress on others (42). The findings of this study corroborate this theory: perceived age discrimination frequently prompts older adults in care facilities to develop more negative self-aging perceptions. This hinders psychological adaptive development and significantly increases the risk of suicidal ideation and even suicidal behavior (8, 21).

### 4.5 The serial mediating role of rumination and self-perceived aging in the association between perceived ageism and suicidal ideation among institutionalized older adults

Additionally, the findings reveal that rumination and perceived self-aging mediate the relationship between perceived ageism and suicidal ideation among institutionalized older adults. Moreover, the study found that perceived ageism is also indirectly associated with suicidal ideation among this population through a chain mediation effect involving rumination and self-perceived aging. This implies that older adults who adopt rumination as a negative cognitive strategy are more prone to repeatedly dwelling on age discrimination and its adverse consequences. This process amplifies the detrimental effects of discriminatory incidents, leading to worsened self-evaluation and the development of negative perceived self-aging (27). For older adults living in residential care, holding negative aging beliefs tends to foster more pessimistic views about their future, exacerbate difficulties in daily life and interpersonal relationships (25), and intensify negative emotions such as stress, loneliness, anxiety, and depression (39). The long-term accumulation of these factors can trigger suicidal ideation.

## 5 Limitations

The findings of this study hold positive implications for countries experiencing accelerated aging in preventing and intervening in age-related suicidal ideation among institutionalized older adults. It is important to note that this research has the following limitations: First, the cross-sectional survey design may not adequately reveal causal relationships between variables. Future studies could employ experimental or longitudinal designs for further validation among long-term care populations. Second, the use of self-reported questionnaires may introduce bias due to participants’ social desirability effects—particularly in institutional settings where residents may wish to appear compliant or optimistic. Third, although the chained mediation analysis allowed us to examine the sequential mediating roles of rumination and self-perceived aging under the current data constraints, this approach relies on observed composite scores and does not permit a global assessment of the overall theoretical model structure. In contrast, structural equation modeling (SEM) offers a more integrative framework for testing complex psychological pathways. For instance, Liu et al. (43; PMID: 36011822) applied SEM to model multiple core constructs of the Health Belief Model as latent variables and evaluated the overall plausibility of their theoretical pathway using five model fit indices (CFI, GFI, RMSEA, NFI, and χ²/df). Such an approach demonstrates greater capacity for integrating and rigorously testing models involving multiple latent psychological mechanisms among vulnerable older populations. Future research should consider adopting SEM or other integrative modeling frameworks, particularly when incorporating multi-item indicators and longitudinal data from residential care samples, to more robustly elucidate the psychological mechanisms linking perceived ageism to suicidal ideation. Fourth, the sample was drawn exclusively from a city in Northeast China, limiting generalizability to other regions or countries, which requires further validation—especially across diverse institutional care contexts. Fifth, perceived ageism exhibited a zero-inflated distribution in our sample, with 75.8% of participants (n=247) reporting no experience of ageism (PA = 0). This likely reflects the relatively protective social environment of long-term care facilities within the Chinese cultural context, where filial norms and structured eldercare may buffer exposure to overt age discrimination. Importantly, as noted by Hayes (44), indirect effects in regression-based mediation models are driven solely by individuals who exhibit variability in the independent variable; thus, the significant serial mediation effect observed in this study was entirely attributable to the subsample who reported any ageism exposure (n=79). Although this data structure does not compromise the validity of the estimated psychological mechanism, it does constrain statistical power. Future studies could employ targeted sampling strategies (e.g., oversampling individuals with perceived ageism experiences) to enhance the detection and generalizability of risk pathways among high-risk subgroups of institutionalized older adults.

## 6 Conclusions

The chain mediation model constructed in this study provides new evidence regarding the relationship and underlying mechanisms between perceived ageism and suicidal ideation among institutionalized older adults. The results demonstrate that perceived ageism is not only directly and positively associated with suicidal ideation among this population, but also indirectly and positively linked through three distinct pathways: (1) a separate mediation pathway involving rumination, (2) a separate mediation pathway involving self-perceived aging, and (3) a chained mediation pathway in which rumination and self-perceived aging sequentially mediate the association. Based on the study findings, the following recommendations are proposed: At the clinical level, in geriatric mental health care for institutionalized older adults, clinicians are encouraged to integrate evidence-based interventions—such as mindfulness-based stress reduction (MBSR), metacognitive therapy, and cognitive restructuring—into routine practice within long-term care settings. These approaches can help institutionalized older adults: (1) Reframe ageist experiences by reducing rumination and fostering adaptive coping; (2) Transform negative self-perceptions of aging into empowering narratives (e.g., “aging equates with resilience and accumulated knowledge”); (3) Strengthen self-worth through value-affirming activities and opportunities for social contribution. Collectively, these strategies target key mechanisms in the ageism–suicidal ideation pathway specific to institutionalized older adults and offer actionable, context-sensitive tools for suicide prevention in residential care environments.

## Data Availability

The raw data supporting the conclusions of this article will be made available by the authors, without undue reservation.

## References

[1] National Bureau of Statistics of China . The Seventh National Population Census (2021). Available online at: https://www.stats.gov.cn/sj/sjjd/202302/t20230202_1896484.html (Accessed June 18, 2024).

[2] World Health Organization . Suicide worldwide in 2019 (2019). Available online at: http://www.who.int/mental_health/suicide-prevention/en/ (Accessed June 18, 2024).

[3] Simon M Chang ES Zeng P Dong X . Prevalence of suicidal ideation, attempts, and completed suicide rate in Chinese aging populations: A systematic review. Arch Gerontology Geriatrics. (2013) 57:250–6. doi: 10.1016/j.archger.2013.05.006. PMID: 23791030 PMC3750072

[4] Liang YJ Deng F Liang P Zhong BL . Suicidal ideation and mental health help-seeking behaviors among older Chinese adults during the COVID-19 pandemic. J Geriatric Psychiatry Neurol. (2022) 35:245–51. doi: 10.1177/08919887221078568. PMID: 35139677 PMC8844439

[5] Wu Y Su B Zhao Y Chen C Zhong P Zheng X . Epidemiological features of suicidal ideation among the elderly in China based meta-analysis. BMC Psychiatry. (2024) 24:562. doi: 10.1186/s12888-024-06010-9. PMID: 39154000 PMC11330032

[6] Nock MK Borges G Bromet EJ Alonso J Angermeyer M Beautrais A . Cross-national prevalence and risk factors for suicidal ideation, plans and attempts. Br J Psychiatry. (2008) 192:98–105. doi: 10.1016/s0084-3970(08)79116-3. PMID: 18245022 PMC2259024

[7] Rothermund K Klusmann V Zacher H . Age discrimination in the context of motivation and healthy aging. Journals Gerontology: Ser B. (2021) 76:S167–80. doi: 10.1093/geronb/gbab081. PMID: 34515776

[8] Kim H Thyer BA Munn JC . The relationship between perceived ageism and depressive symptoms in later life: Understanding the mediating effects of self-perception of aging and purpose in life, using structural equation modeling. Educ Gerontology. (2019) 45:105–19. doi: 10.1080/03601277.2019.1583403. PMID: 41735180

[9] Wang X Wang Y Zhang F Ge D Guo Z . Associations between social isolation, perceived ageism and subjective well-being among rural Chinese older adults: A cross-sectional study. Geriatric Nurs. (2024) 59:598–603. doi: 10.1016/j.gerinurse.2024.08.014. PMID: 39178626

[10] Garstka TA Schmitt MT Branscombe NR Hummert ML . How young and older adults differ in their responses to perceived age discrimination. Psychol Aging. (2004) 19:326. doi: 10.1037/0882-7974.19.2.326. PMID: 15222826

[11] Kessler RC Mickelson KD Williams DR . The prevalence, distribution, and mental health correlates of perceived discrimination in the United States. J Health Soc Behav. (1999) 40:208–30. doi: 10.2307/2676349. PMID: 10513145

[12] Browning SD Penning MJ Wu Z . Perceived age discrimination: Implications for mental health and life satisfaction in middle and later life—a research note. Can Stud Popul. (2020) 47:245–62. doi: 10.1007/s42650-020-00035-7. PMID: 41776007

[13] Ko Y Han SY Jang HY . Factors influencing suicidal ideation and attempts among older Korean adults: Focusing on age discrimination and neglect. Int J Environ Res Public Health. (2021) 18:1852. doi: 10.3390/ijerph18041852. PMID: 33672881 PMC7917585

[14] Gendron T Camp A Amateau G Iwanaga K . Internalized ageism as a risk factor for suicidal ideation in later life. Aging Ment Health. (2024) 28:701–5. doi: 10.1080/13607863.2023.2271870. PMID: 37861403

[15] Luo J Wong NM Zhang R Wu J Shao R Chan CC . A network analysis of rumination on loneliness and the relationship with depression. Nat Ment Health. (2025) 3:46–57. doi: 10.1038/s44220-024-00350-x. PMID: 41771954

[16] Otto MW Lubin RE Rosenfield D Taylor DJ Birk JL Espie CA . The association between race-and ethnicity-related stressors and sleep: The role of rumination and anxiety sensitivity. Sleep. (2022) 45:zsac117. doi: 10.1093/sleep/zsac117. PMID: 35639820 PMC9548665

[17] Wang SB Borders A . Rumination mediates the associations between sexual minority stressors and disordered eating, particularly for men. Eating Weight Disorders-Studies Anorexia Bulimia Obes. (2017) 22:699–706. doi: 10.1007/s40519-016-0350-0. PMID: 28039668

[18] Lin L Liu J Yang Y Liu Y Wang C Liu T . The impact of negative life events in college students' suicidal ideation: Mediating role of rumination and moderating role of dispositional optimism. Stud Psychol Behav. (2019) 17:569. Available online at: https://psybeh.tjnu.edu.cn/EN/Y2019/V17/I4/569 (Accessed June 20, 2024).

[19] Le GH Wong S Au H Badulescu S Gill H Vasudeva S . Association between rumination, suicidal ideation and suicide attempts in persons with depressive and other mood disorders and healthy controls: A systematic review and meta-analysis. J Affect Disord. (2025) 368:513–27. doi: 10.1016/j.jad.2024.09.118. PMID: 39303880

[20] Miranda R Polanco-Roman L Tsypes A Valderrama J . Perceived discrimination, ruminative subtypes, and risk for depressive symptoms in emerging adulthood. Cultural Diversity Ethnic Minority Psychol. (2013) 19:395. doi: 10.1037/a0033504. PMID: 24188536 PMC6002758

[21] Han J Richardson VE . The relationships among perceived discrimination, self-perceptions of aging, and depressive symptoms: A longitudinal examination of age discrimination. Aging Ment Health. (2015) 19:747–55. doi: 10.1080/13607863.2014.962007. PMID: 25266167

[22] Levy B . Stereotype embodiment: A psychosocial approach to aging. Curr Dir psychol Sci. (2009) 18:332–6. doi: 10.1111/j.1467-8721.2009.01662.x. PMID: 20802838 PMC2927354

[23] Coudin G Alexopoulos T . Help me! I’m old!” How negative aging stereotypes create dependency among older adults. Aging Ment Health. (2010) 14:516–23. doi: 10.1080/13607861003713182. PMID: 20480414

[24] Marquet M Chasteen AL Plaks JE Balasubramaniam L . Understanding the mechanisms underlying the effects of negative age stereotypes and perceived age discrimination on older adults’ well-being. Aging Ment Health. (2019) 23:1666–73. doi: 10.1080/13607863.2018.1514487. PMID: 30457350

[25] Freeman AT Santini ZI Tyrovolas S Rummel-Kluge C Haro JM Koyanagi A . Negative perceptions of ageing predict the onset and persistence of depression and anxiety: Findings from a prospective analysis of the Irish Longitudinal Study on Ageing (TILDA). J Affect Disord. (2016) 199:132–8. doi: 10.1016/j.jad.2016.03.042. PMID: 27104801

[26] Vauclair CM Lima ML Abrams D Swift HJ Bratt C . What do older people think that others think of them, and does it matter? The role of meta-perceptions and social norms in the prediction of perceived age discrimination. Psychol Aging. (2016) 31:699. doi: 10.1037/pag0000125. PMID: 27831711 PMC5104248

[27] Dutt AJ Wahl HW Rupprecht FS . Mindful vs. mind full: Processing strategies moderate the association between subjective aging experiences and depressive symptoms. Psychol Aging. (2018) 33:630. doi: 10.1037/pag0000245. PMID: 29708386

[28] Han X Yang HF . Pilot study of the Nolen-Hoeksema rumination scale in China. Chin J Clin Psychol. (2009) 17:550–1.

[29] Kothari MM Murali MR Ganu MA . The effect of rumination on wellbeing among geriatric population. In: In-Quest: Students' Research Compendium 2022. Smt. Maniben M. P. Shah Women's College of Arts and Commerce; 2022. p. 81–96.

[30] Hu N Meng L Liu K . Study on the reliability and validity of brief ageing perceptions questionnaire among the community elderly. Modern Prev Med. (2018) 45:655–8.

[31] Zhang ZJ . Handbook of Behavioral Medicine Scales, vol. 1. Beijing: Chinese Medical Electronic Audiovisual Press (2005).

[32] Cheng JC Xing FM Xiahou WX Yu LS Tang HY . Status quo and influencing factors of aging expectations among community-dwelling elderly. J Nurs Sci. (2021) 36:15–8.

[33] Tan QY Deng YX Ji WQ Wang DH . Chain mediating effect of self-stereotyping and subjective memory complaints on the relationship between self-perceived aging and dementia fear among rural older adults. J Nurs. (2025) 32:56–62. doi: 10.16460/j.issn1008-9969.2025.01.056

[34] Luo YY Du YS Yao GY Zhang JH Zhang HM Qin LW . Construction of a structural equation model on motor function, self-perception of aging, and depression among community-dwelling elderly. J Nurs Sci. (2021) 36:80–3.

[35] Fu LT Zhang XH Sun X . Mediating effects of perceived stress and perceived social support on the relationship between perceived age discrimination and suicidal ideation among older adults in nursing homes. J Nurs. (2023) 30:1–5. doi: 10.16460/j.issn1008-9969.2023.20.001

[36] Kim G Lee MA . Age discrimination and suicidal ideation among Korean older adults. Am J Geriatric Psychiatry. (2020) 28:748–54. doi: 10.1016/j.jagp.2019.12.002. PMID: 31926841

[37] Kim HHS Jung JH . Ageism, religiosity, and wellbeing among older adults: Evidence from the European Social Survey (ESS4). Res Aging. (2021) 43:214–26. doi: 10.1177/0164027520953632. PMID: 32873186

[38] Borders A Liang CT . Rumination partially mediates the associations between perceived ethnic discrimination, emotional distress, and aggression. Cultural Diversity Ethnic Minority Psychol. (2011) 17:125. doi: 10.1037/a0023305. PMID: 21604836

[39] Vogt Yuan AS . Perceived age discrimination and mental health. Soc Forces. (2007) 86:291–311. doi: 10.1353/sof.2007.0113. PMID: 34409987

[40] Van Orden K Deming C . Late-life suicide prevention strategies: Current status and future directions. Curr Opin Psychol. (2018) 22:79–83. doi: 10.1016/j.copsyc.2017.08.033. PMID: 28938218 PMC5843499

[41] Cooley CH . Human nature and the social order. Routledge (2017).

[42] Van Orden KA Witte TK Cukrowicz KC Braithwaite SR Selby EA Joiner TE . The interpersonal theory of suicide. psychol Rev. (2010) 117:575. doi: 10.1037/a0018697. PMID: 20438238 PMC3130348

[43] Liu H Lai G Shi G Zhong X . The influencing factors of HIV-preventive behavior based on health belief model among hiv-negative msms in Western China: A structural equation modeling analysis. Int J Environ Res Public Health. (2022) 19:10185. doi: 10.3390/ijerph191610185. PMID: 36011822 PMC9407807

[44] Hayes AF . Introduction to mediation, moderation, and conditional process analysis: A regression-based approach. 3rd ed. Guilford Press (2022) p. 89–128.

