# Supplementary material for: Perceived ageism and suicidal ideation among Chinese older adults: the mediating role of rumination and self-perceived aging
Source: Front Psychiatry. 2026 Apr 10;17:1747979. doi: 10.3389/fpsyt.2026.1747979 (PMC13106181; doi:10.3389/fpsyt.2026.1747979)
Supplement: Supplementary file 1 [file DataSheet1.pdf]

Appendix A:

1. **Perceived Ageism Scale (PAS)**

This study adopted the assessment method from the European Ageism Prevalence Survey (Vauclair et al., 2016). It comprises 3 items scored on a 5-point scale (0=Never ~ 4=Always), with higher scores indicating greater perceived ageism. The internal consistency alpha coefficient for this study's scale was 0.875.

Instructions: Please read the following statements carefully and respond according to your actual situation. Circle the number corresponding to the option that best fits your response for each item.

Item Number	Statement	Never	Seldom	Some times	Often	Always
1	In the past year, have you felt that someone showed prejudice or treated you unfairly because of your age?	0	1	2	3	4
2	In the past year, have you felt that someone showed a lack of respect toward you because of your age, such as ignoring or looking down on you?	0	1	2	3	4
3	In the past year, has someone treated you very poorly because of your age, for example, by insulting you, mistreating you, or denying you services?	0	1	2	3	4

Vauclair, C. M., Lima, M. L., Abrams, D., Swift, H. J., & Bratt, C. (2016). What do older people think that others think of them, and does it matter? The role of meta-perceptions and social norms in the prediction of perceived age discrimination.

Psychology and Aging,31(7), 699. doi:10.1037/pag0000125

2. Ruminative Responses Scale (RRS)

This scale was introduced to China by Han and Yang (2009). It comprises 22 items across three factors (symptomatic rumination, obsessive thinking, and reflective rumination), scored on a 4-point scale (1=sometimes to 4=always). Higher scores indicate greater ruminative thinking. The internal consistency alpha coefficient for this study was 0.952.

Instructions: Everyone experiences some degree of distress from time to time, and may engage in different thoughts or behaviors as a result. Please imagine how you generally think or act when you feel distressed (not what you think you *should* do, but what you *typically* do). For each statement below, indicate how often you engage in the thought or behavior by checking (√) the corresponding number (1 = Never, 2 = Sometimes, 3 = Often, 4 = Always). Please note that there are no right or wrong answers—the options reflect frequency, not value judgments.

**Item Number**	**Statement**	**Never**	**Some times**	**Often**	**Always**
1	I often think about how lonely I am.	1	2	3	4
2	I often think, “If I can’t stop thinking about this, I won’t be able to focus on what I need to do.”	1	2	3	4
3	I often think about my feelings of fatigue and distress.	1	2	3	4

4	I often think, “How hard it is to concentrate.”	1	2	3	4
5	I often think about what I might have done to cause this.	1	2	3	4
6	I often think about how passive and unmotivated I am.	1	2	3	4
7	I often analyze recent events to try to understand why I feel distressed.	1	2	3	4
8	I often think about how numb I feel to other things.	1	2	3	4
9	I often think about why things always go wrong for me.	1	2	3	4
10	I often think, “Why do I always end up like this?”	1	2	3	4
11	I often think alone about why this is happening.	1	2	3	4

12	I often write down what I am thinking and analyze it.	1	2	3	4
13	I often think about the current situation and wish it would improve.	1	2	3	4
14	I often think that if this feeling continues, I won't be able to concentrate.	1	2	3	4
15	I often wonder why I have these problems while others don't.	1	2	3	4
16	I often think about why I can't handle things better.	1	2	3	4
17	I often wonder why I feel so sad.	1	2	3	4
18	I often think about my shortcomings, failures, mistakes, and faults.	1	2	3	4
19	I often think, "I don't feel like doing anything."	1	2	3	4

20	I often analyze my personality to understand why I feel depressed.	1	2	3	4
21	I often go somewhere alone to think about my feelings.	1	2	3	4
22	I often think about how angry I am with myself.	1	2	3	4

Han, X., & Yang, H. F. (2009). Pilot study of the Nolen-Hoeksema Rumination Scale in China. Chinese Journal of Clinical Psychology,17(5), 550-551, 549.

3. Brief Ageing Perceptions Questionnaire (B-APQ)

This scale was revised by Hu et al. (2018). It comprises 17 items across 5 factors (Chronic Time, Positive Outcomes, Positive Control, Negative Outcomes and Control, Emotional Representation) using a 5-point scale (1=Strongly Disagree ~ 5=Strongly Agree). Higher scores indicate more negative self-perceived aging. The internal consistency alpha coefficient for this study was 0.802.

Instructions: Please rate the following statements based on your actual feelings and experiences, and circle the corresponding number for each option.

Item Number	Statement	Strongly Disagree	Somewhat Disagree	Neither Agree nor Disagree	Somewhat Agree	Strongly Agree
1	I always categorize myself as an elderly person.	1	2	3	4	5

2	I am constantly aware of the fact that I am aging.	1	2	3	4	5
3	In everything I do, I can feel my age.	1	2	3	4	5
4	As I age, I become wiser.	1	2	3	4	5
5	As I age, I continue to grow.	1	2	3	4	5
6	As I age, I learn to cherish things more.	1	2	3	4	5
7	It saddens me to think that aging may affect what I can do.	1	2	3	4	5
8	The quality of my social life in later years depends on me.	1	2	3	4	5
9	The quality of my relationships in later years depends on me.	1	2	3	4	5

10	Whether I can continue to lead a fulfilling life is up to me.	1	2	3	4	5
11	Aging may make me increasingly dependent.	1	2	3	4	5
12	As I age, I can participate in fewer activities.	1	2	3	4	5
13	As I age, I may not handle problems as well as before.	1	2	3	4	5
14	The slowing down due to aging is beyond my control.	1	2	3	4	5
15	The impact of aging on my social life is something I cannot control.	1	2	3	4	5
16	I worry that aging will affect my relationships.	1	2	3	4	5
17	The thought of aging makes me feel angry.	1	2	3	4	5

Hu, N., Meng, L., & Liu, K. (2018). Study on the reliability and validity of brief ageing perceptions questionnaire among the community elderly. *Modern Preventive Medicine*,*45*(4), 655-658.

4. Self-rating Idea of Suicide Scale (SIOSS)

This scale was developed by Wang et al. (1999). It consists of 26 items across 4 factors (Despair, Optimism, Sleep, and Concealment), scored on a 2-point scale (Yes =1 point, No=0 points). The total suicide ideation score is the sum of the Despair, Optimism, and Sleep factor scores. A higher total score indicates stronger suicidal ideation. In this study, the internal consistency coefficient for this scale was 0.856.

Instructions: This questionnaire contains 26 questions. Please read each item carefully, make sure you understand it, and then mark "Yes" or "No" under each statement based on your actual situation. Answer every question. There is no time limit, but please do not take too long.

Item Number	Statement	Yes	No
1	My daily life is filled with things that interest me.		
2	I am convinced that life is harsh to me.		
3	I often feel pessimistic and hopeless.		

4	I tend to cry or feel like crying easily.		
5	I fall asleep easily and sleep well through the night.		
6	Sometimes I tell lies.		
7	How wonderful it is to live in such a diverse and vibrant era.		
8	I truly lack self-confidence.		
9	I sometimes lose my temper.		
10	I always feel that life is valuable.		
11	Most of the time, I think it would be better if I were dead.		

12	I sleep restlessly and wake up easily.		
13	Sometimes I gossip about others.		
14	Sometimes I feel utterly useless.		
15	Occasionally, I laugh at vulgar jokes.		
16	My future seems hopeless.		
17	I want to end my own life.		
18	I wake up too early.		
19	I feel that my life is a failure.		

20	I always take things too seriously.		
21	I have hope for the future.		
22	I have attempted suicide before.		
23	Sometimes I feel like I am about to break down.		
24	There are times when I suffer from insomnia due to worries.		
25	I have damaged or lost things belonging to others.		
26	Sometimes I wish to die, but I feel deeply conflicted about it.		

Wang, X. D., Wang, X. L., & Ma, H. (1999). *Rating scales for mental health*. Chinese Mental Health Journal Publisher. (in Chinese)
